# Bis(4-hydroxy­pyridinium) sulfate monohydrate

**DOI:** 10.1107/S1600536809048521

**Published:** 2009-11-21

**Authors:** Ying-Ming Xu, Shan Gao, Seik Weng Ng

**Affiliations:** a College of Chemistry and Materials Science, Heilongjiang University, Harbin 150080, People’s Republic of China; b Department of Chemistry, University of Malaya, 50603 Kuala Lumpur, Malaysia

## Abstract

In the crystal structure of the title salt, 2C_5_H_6_NO^+^·SO_4_^2−^·H_2_O, one planar (r.m.s. deviation = 0.01 Å) cation is stacked approximately over the other [dihedral angle between planes = 8.6 (1)°]. The pyridinium and hydr­oxy H atoms are hydrogen-bond donor atoms to the O atoms of the sulfate anion; the cations, anions and water mol­ecules are consolidated into a three-dimensional network through O—H⋯O and N—H⋯O hydrogen bonds.

## Related literature

For the crystal structures of 4-hydroxy­pyridinium salts, see: Fukunaga *et al.* (2004 ▶); Gao *et al.* (2004 ▶); Kiviniemi *et al.* (2001 ▶); Wang *et al.* (2006 ▶).
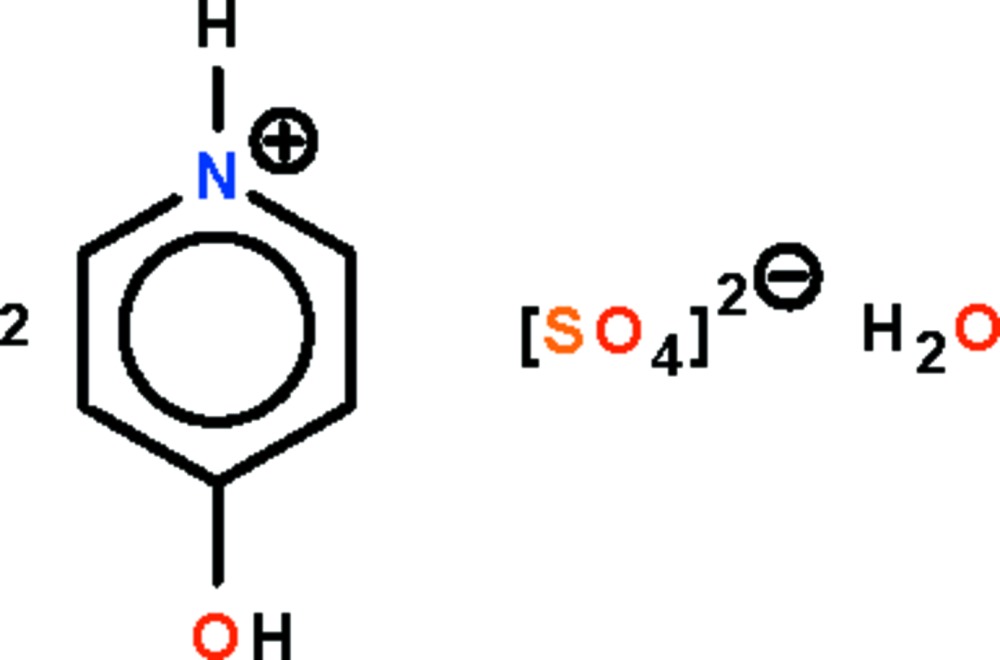


## Experimental

### 

#### Crystal data

2C_5_H_6_NO^+^·SO_4_^2−^·H_2_O
*M* *_r_* = 306.29
Monoclinic, 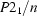

*a* = 7.1404 (2) Å
*b* = 19.9797 (5) Å
*c* = 9.5148 (2) Å
β = 102.557 (1)°
*V* = 1324.94 (6) Å^3^
*Z* = 4
Mo *K*α radiation
μ = 0.28 mm^−1^
*T* = 293 K
0.25 × 0.18 × 0.16 mm

#### Data collection

Rigaku R-AXIS RAPID IP diffractometer
Absorption correction: multi-scan (*ABSCOR*; Higashi, 1995 ▶) *T*_min_ = 0.934, *T*_max_ = 0.957
12868 measured reflections
3032 independent reflections
2693 reflections with *I* > 2σ(*I*)
*R*_int_ = 0.020

#### Refinement

*R*[*F* ^2^ > 2σ(*F* ^2^)] = 0.034
*wR*(*F* ^2^) = 0.103
*S* = 1.05
3032 reflections
197 parameters
6 restraints
H atoms treated by a mixture of independent and constrained refinement
Δρ_max_ = 0.42 e Å^−3^
Δρ_min_ = −0.43 e Å^−3^

### 

Data collection: *RAPID-AUTO* (Rigaku, 1998 ▶); cell refinement: *RAPID-AUTO*; data reduction: *CrystalClear* (Rigaku/MSC, 2002 ▶); program(s) used to solve structure: *SHELXS97* (Sheldrick, 2008 ▶); program(s) used to refine structure: *SHELXL97* (Sheldrick, 2008 ▶); molecular graphics: *X-SEED* (Barbour, 2001 ▶); software used to prepare material for publication: *publCIF* (Westrip, 2009 ▶).

## Supplementary Material

Crystal structure: contains datablocks global, I. DOI: 10.1107/S1600536809048521/xu2675sup1.cif

Structure factors: contains datablocks I. DOI: 10.1107/S1600536809048521/xu2675Isup2.hkl

Additional supplementary materials:  crystallographic information; 3D view; checkCIF report

## Figures and Tables

**Table 1:** Hydrogen-bond geometry (Å, °)

*D*—H⋯*A*	*D*—H	H⋯*A*	*D*⋯*A*	*D*—H⋯*A*
O5—H5⋯O1w	0.85 (1)	1.71 (1)	2.552 (2)	171 (2)
O6—H6⋯O2	0.86 (1)	1.68 (1)	2.539 (1)	177 (2)
O1w—H11⋯O1	0.84 (1)	1.93 (1)	2.765 (2)	170 (3)
O1w—H12⋯O3^i^	0.85 (1)	1.99 (2)	2.783 (2)	157 (3)
N1—H1n⋯O4^ii^	0.86	1.95	2.766 (2)	158
N2—H2n⋯O3^iii^	0.86	1.87	2.705 (2)	163

Symmetry codes: (i) 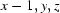; (ii) 
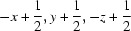
; (iii) 
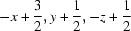
.

**Fig. 1.**
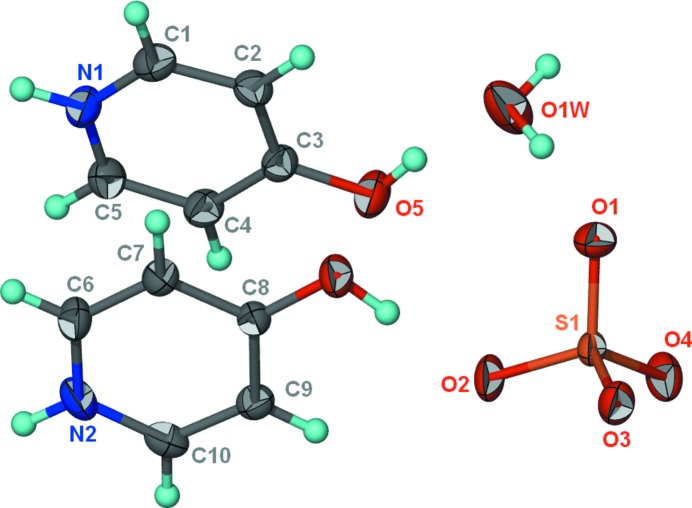
Thermal ellipsoid plot (Barbour, 2001) of 2[C5H6NO][SO4].H2O at the 50% probability level; hydrogen atoms are drawn as spheres of arbitrary radius.

## supplementary crystallographic information

### Experimental

Copper nitrate (0.37 g, 2 mmol) and 4-hydroxypyridine-3-sulfonic acid (0.35 g, 2 mmol) were dissolved in hot water. The pH value was adjusted to 6 with 0.1 *M* sodium hydroxide. The solution was allowed to evaporate slowly at room temperature; colorless prismatic crystals were isolated from the blue-green solution after several days.

### Refinement

Carbon-bound H-atoms were placed in calculated positions (C–H 0.93 Å) and were included in the refinement in the riding model approximation, with *U*(H) set to 1.2*U*(C). The water H-atoms were located in a difference Fourier map, and were refined with a distance restraint of O–H = 0.85±0.01 Å; their temperature factors were refined. The pyridinium H-atoms could be found in a difference Fourier map; however, their refinement led to somewhat unsatisfactory angles. As such, their positions were fixed and their temperatures tied to those of the parent atoms.

### Crystal data

**Table:**

2C_5_H_6_NO^+^·SO_4_^2^^−^·H_2_O	*F*(000) = 640
*M**_r_* = 306.29	*D*_x_ = 1.535 Mg m^−^^3^
Monoclinic, *P*2_1_/*n*	Mo *K*α radiation, λ = 0.71073 Å
Hall symbol: -P 2yn	Cell parameters from 11052 reflections
*a* = 7.1404 (2) Å	θ = 3.0–27.4°
*b* = 19.9797 (5) Å	µ = 0.28 mm^−^^1^
*c* = 9.5148 (2) Å	*T* = 293 K
β = 102.557 (1)°	Prism, colorless
*V* = 1324.94 (6) Å^3^	0.25 × 0.18 × 0.16 mm
*Z* = 4	

### Data collection

**Table:**

Rigaku R-AXIS RAPID IP diffractometer	3032 independent reflections
Radiation source: fine-focus sealed tube	2693 reflections with *I* > 2σ(*I*)
graphite	*R*_int_ = 0.020
ω scan	θ_max_ = 27.4°, θ_min_ = 3.0°
Absorption correction: multi-scan (*ABSCOR*; Higashi, 1995)	*h* = −9→9
*T*_min_ = 0.934, *T*_max_ = 0.957	*k* = −25→25
12868 measured reflections	*l* = −12→12

### Refinement

**Table:**

Refinement on *F*^2^	Primary atom site location: structure-invariant direct methods
Least-squares matrix: full	Secondary atom site location: difference Fourier map
*R*[*F*^2^ > 2σ(*F*^2^)] = 0.034	Hydrogen site location: inferred from neighbouring sites
*wR*(*F*^2^) = 0.103	H atoms treated by a mixture of independent and constrained refinement
*S* = 1.05	*w* = 1/[σ^2^(*F*_o_^2^) + (0.0703*P*)^2^ + 0.2237*P*] where *P* = (*F*_o_^2^ + 2*F*_c_^2^)/3
3032 reflections	(Δ/σ)_max_ = 0.001
197 parameters	Δρ_max_ = 0.42 e Å^−^^3^
6 restraints	Δρ_min_ = −0.43 e Å^−^^3^

### Fractional atomic coordinates and isotropic or equivalent isotropic displacement parameters (Å^2^)

**Table:**

	*x*	*y*	*z*	*U*_iso_*/*U*_eq_	
S1	0.61983 (4)	0.073598 (14)	0.24878 (3)	0.02657 (12)	
O1	0.43221 (15)	0.06385 (5)	0.15168 (11)	0.0397 (3)	
O2	0.64861 (17)	0.14531 (5)	0.28579 (12)	0.0436 (3)	
O3	0.77259 (15)	0.05185 (5)	0.17503 (12)	0.0388 (2)	
O4	0.63392 (17)	0.03440 (5)	0.38109 (10)	0.0417 (3)	
O5	0.20084 (17)	0.22956 (5)	0.36294 (12)	0.0425 (3)	
O6	0.47857 (16)	0.23613 (5)	0.11899 (11)	0.0373 (2)	
O1W	0.1093 (2)	0.12590 (7)	0.2064 (2)	0.0645 (4)	
N1	0.04132 (18)	0.41179 (6)	0.18890 (13)	0.0356 (3)	
H1N	0.0088	0.4507	0.1532	0.043*	
N2	0.62608 (18)	0.42205 (6)	0.27653 (15)	0.0379 (3)	
H2N	0.6556	0.4617	0.3091	0.045*	
C1	−0.0033 (2)	0.35736 (7)	0.10572 (15)	0.0355 (3)	
H1	−0.0686	0.3623	0.0105	0.043*	
C2	0.0458 (2)	0.29479 (7)	0.15884 (14)	0.0321 (3)	
H2	0.0135	0.2573	0.1006	0.039*	
C3	0.14570 (19)	0.28776 (7)	0.30255 (14)	0.0304 (3)	
C4	0.1913 (2)	0.34564 (7)	0.38645 (14)	0.0340 (3)	
H4	0.2585	0.3424	0.4816	0.041*	
C5	0.1361 (2)	0.40666 (7)	0.32736 (16)	0.0358 (3)	
H5A	0.1643	0.4451	0.3831	0.043*	
C6	0.5135 (2)	0.41430 (7)	0.14439 (17)	0.0382 (3)	
H6A	0.4688	0.4519	0.0897	0.046*	
C7	0.4642 (2)	0.35222 (7)	0.08949 (14)	0.0329 (3)	
H7	0.3870	0.3474	−0.0022	0.039*	
C8	0.53127 (18)	0.29565 (6)	0.17300 (13)	0.0271 (3)	
C9	0.64828 (19)	0.30528 (7)	0.31067 (14)	0.0304 (3)	
H9	0.6946	0.2688	0.3685	0.036*	
C10	0.6930 (2)	0.36886 (8)	0.35840 (15)	0.0350 (3)	
H10	0.7712	0.3755	0.4491	0.042*	
H5	0.158 (3)	0.1970 (8)	0.308 (2)	0.060 (6)*	
H6	0.540 (3)	0.2060 (8)	0.1754 (18)	0.053 (5)*	
H11	0.204 (3)	0.1028 (11)	0.195 (3)	0.076 (7)*	
H12	0.021 (3)	0.1002 (12)	0.221 (3)	0.093 (9)*	

### Atomic displacement parameters (Å^2^)

**Table:**

	*U*^11^	*U*^22^	*U*^33^	*U*^12^	*U*^13^	*U*^23^
S1	0.03062 (19)	0.01762 (17)	0.02844 (18)	0.00117 (10)	−0.00024 (13)	−0.00033 (10)
O1	0.0326 (5)	0.0376 (5)	0.0431 (6)	0.0009 (4)	−0.0045 (4)	−0.0053 (4)
O2	0.0599 (7)	0.0181 (5)	0.0440 (6)	0.0024 (4)	−0.0081 (5)	−0.0031 (4)
O3	0.0371 (5)	0.0294 (5)	0.0513 (6)	0.0046 (4)	0.0123 (5)	0.0042 (4)
O4	0.0610 (7)	0.0301 (5)	0.0308 (5)	−0.0052 (5)	0.0030 (4)	0.0034 (4)
O5	0.0543 (7)	0.0295 (5)	0.0406 (6)	0.0081 (5)	0.0032 (5)	0.0031 (4)
O6	0.0479 (6)	0.0239 (5)	0.0354 (5)	−0.0011 (4)	−0.0010 (4)	−0.0020 (4)
O1W	0.0465 (7)	0.0422 (7)	0.1120 (11)	−0.0086 (6)	0.0328 (8)	−0.0245 (7)
N1	0.0367 (6)	0.0292 (6)	0.0408 (6)	0.0047 (5)	0.0086 (5)	0.0058 (5)
N2	0.0384 (7)	0.0271 (6)	0.0497 (7)	−0.0089 (5)	0.0132 (5)	−0.0081 (5)
C1	0.0324 (7)	0.0414 (8)	0.0317 (6)	0.0018 (6)	0.0045 (5)	0.0033 (5)
C2	0.0316 (7)	0.0323 (6)	0.0319 (6)	−0.0015 (5)	0.0056 (5)	−0.0045 (5)
C3	0.0282 (6)	0.0295 (6)	0.0341 (6)	0.0034 (5)	0.0085 (5)	0.0016 (5)
C4	0.0347 (7)	0.0348 (7)	0.0309 (6)	0.0027 (5)	0.0034 (5)	−0.0028 (5)
C5	0.0369 (7)	0.0305 (6)	0.0396 (7)	0.0012 (6)	0.0077 (6)	−0.0039 (6)
C6	0.0403 (8)	0.0264 (6)	0.0481 (8)	0.0004 (6)	0.0099 (6)	0.0078 (6)
C7	0.0333 (7)	0.0308 (6)	0.0321 (6)	0.0001 (5)	0.0017 (5)	0.0050 (5)
C8	0.0257 (6)	0.0246 (6)	0.0309 (6)	−0.0005 (4)	0.0060 (5)	0.0000 (5)
C9	0.0285 (6)	0.0323 (6)	0.0294 (6)	0.0011 (5)	0.0042 (5)	0.0022 (5)
C10	0.0288 (6)	0.0408 (7)	0.0350 (7)	−0.0065 (5)	0.0055 (5)	−0.0072 (6)

### Geometric parameters (Å, °)

**Table:**

S1—O4	1.4674 (10)	C1—C2	1.365 (2)
S1—O1	1.4656 (10)	C1—H1	0.9300
S1—O2	1.4790 (10)	C2—C3	1.4051 (18)
S1—O3	1.4845 (10)	C2—H2	0.9300
O5—C3	1.3187 (16)	C3—C4	1.4026 (19)
O5—H5	0.849 (9)	C4—C5	1.364 (2)
O6—C8	1.3175 (15)	C4—H4	0.9300
O6—H6	0.861 (9)	C5—H5A	0.9300
O1W—H11	0.844 (10)	C6—C7	1.362 (2)
O1W—H12	0.847 (10)	C6—H6A	0.9300
N1—C1	1.3419 (19)	C7—C8	1.4046 (18)
N1—C5	1.3479 (19)	C7—H7	0.9300
N1—H1N	0.8600	C8—C9	1.4054 (18)
N2—C10	1.343 (2)	C9—C10	1.3633 (19)
N2—C6	1.346 (2)	C9—H9	0.9300
N2—H2N	0.8600	C10—H10	0.9300
			
O4—S1—O1	110.65 (7)	C4—C3—C2	118.51 (12)
O4—S1—O2	109.43 (6)	C5—C4—C3	119.46 (12)
O1—S1—O2	109.85 (6)	C5—C4—H4	120.3
O4—S1—O3	109.21 (6)	C3—C4—H4	120.3
O1—S1—O3	109.15 (6)	N1—C5—C4	120.62 (13)
O2—S1—O3	108.53 (7)	N1—C5—H5A	119.7
C3—O5—H5	112.0 (15)	C4—C5—H5A	119.7
C8—O6—H6	109.0 (14)	N2—C6—C7	121.01 (13)
H11—O1W—H12	109 (3)	N2—C6—H6A	119.5
C1—N1—C5	121.28 (12)	C7—C6—H6A	119.5
C1—N1—H1N	119.4	C6—C7—C8	119.20 (13)
C5—N1—H1N	119.4	C6—C7—H7	120.4
C10—N2—C6	121.06 (12)	C8—C7—H7	120.4
C10—N2—H2N	119.5	O6—C8—C7	118.19 (12)
C6—N2—H2N	119.5	O6—C8—C9	123.28 (11)
N1—C1—C2	120.98 (13)	C7—C8—C9	118.52 (12)
N1—C1—H1	119.5	C10—C9—C8	119.13 (12)
C2—C1—H1	119.5	C10—C9—H9	120.4
C1—C2—C3	119.14 (12)	C8—C9—H9	120.4
C1—C2—H2	120.4	N2—C10—C9	121.07 (12)
C3—C2—H2	120.4	N2—C10—H10	119.5
O5—C3—C4	117.95 (12)	C9—C10—H10	119.5
O5—C3—C2	123.54 (12)		
			
C5—N1—C1—C2	0.1 (2)	C10—N2—C6—C7	−0.1 (2)
N1—C1—C2—C3	−0.5 (2)	N2—C6—C7—C8	0.4 (2)
C1—C2—C3—O5	−179.30 (13)	C6—C7—C8—O6	178.62 (13)
C1—C2—C3—C4	0.1 (2)	C6—C7—C8—C9	−0.3 (2)
O5—C3—C4—C5	−179.92 (13)	O6—C8—C9—C10	−179.00 (12)
C2—C3—C4—C5	0.7 (2)	C7—C8—C9—C10	−0.1 (2)
C1—N1—C5—C4	0.7 (2)	C6—N2—C10—C9	−0.4 (2)
C3—C4—C5—N1	−1.1 (2)	C8—C9—C10—N2	0.5 (2)

### Hydrogen-bond geometry (Å, °)

**Table:**

*D*—H···*A*	*D*—H	H···*A*	*D*···*A*	*D*—H···*A*
O5—H5···O1w	0.85 (1)	1.71 (1)	2.552 (2)	171 (2)
O6—H6···O2	0.86 (1)	1.68 (1)	2.539 (1)	177 (2)
O1w—H11···O1	0.84 (1)	1.93 (1)	2.765 (2)	170 (3)
O1w—H12···O3^i^	0.85 (1)	1.99 (2)	2.783 (2)	157 (3)
N1—H1n···O4^ii^	0.86	1.95	2.766 (2)	158
N2—H2n···O3^iii^	0.86	1.87	2.705 (2)	163

Symmetry codes: (i) *x*−1, *y*, *z*; (ii) −*x*+1/2, *y*+1/2, −*z*+1/2; (iii) −*x*+3/2, *y*+1/2, −*z*+1/2.
